# The diurnal emission of floral scent in *Oncidium* hybrid orchid is controlled by *CIRCADIAN CLOCK ASSOCIATED 1* (*CCA1*) through the direct regulation on *terpene synthase*

**DOI:** 10.1186/s12870-022-03850-z

**Published:** 2022-10-04

**Authors:** Chao-Wei Yeh, Hui-Qin Zhong, Yung-Feng Ho, Zhi-Hong Tian, Kai-Wun Yeh

**Affiliations:** 1 Institute of Plant Biology, College of Life Science, National Taiwan University, No 1, Sect. 4, Roosevelt Road, 106 Taipei, Taiwan; 2 Fujian Engineering Research Center for Characteristic Floriculture, Crop Research Institute, Fujian Academy of Agricultural Sciences, Fuzhou, Fujian Province China; 3 Hubei Key Laboratory of Waterlogging Disaster and Agricultural Use of Wetland, College of Life Science, Yangtze University, Jingzhou, 434025 Hubei China; 4 Center for Weather Climate and Disaster Research, National Taiwan University, Taipei, 106 Taiwan

**Keywords:** Floral scent, Circadian rhythm, *Oncidium* Sharry Baby, CIRCADIAN CLOCK ASSOCIATED 1 (CCA1), CCA1 binding site (CBS)

## Abstract

**Background:**

To adapt the periodic fluctuation of environmental factors, plants are subtle to monitor the natural variation for the growth and development. The daily activities and physiological functions in coordination with the natural variation are regulated by circadian clock genes. The circadian emission of floral scents is one of the rhythmic physiological activities controlled by circadian clock genes. Here, we study the molecular mechanism of circadian emission pattern of ocimene and linalool compounds in *Oncidium* Sharry Baby (*Onc*. SB) orchid.

**Results:**

GC-Mass analysis revealed that *Onc*. SB periodically emitted ocimene and linalool during 6 to 14 o’clock daily. *Terpene synthase*, one of the key gene in the terpenoid biosynthetic pathway is expressed in coordination with scent emission. The promoter structure of *terpene synthase* revealed a circadian binding sequence (CBS), 5’-AGATTTTT-3’ for CIRCADIAN CLOCK ASSOCIATED1 (CCA1) transcription factor. EMSA data confirms the binding affinity of CCA1. Transactivation assay further verified that *TPS* expression is regulated by CCA1. It suggests that the emission of floral scents is controlled by CCA1.

**Conclusions:**

The work validates that the mechanism of circadian emission of floral scents in *Onc*. Sharry Baby is controlled by the oscillator gene, *CCA1*(*CIRCADIAN CLOCK ASSOCIATED 1*) under light condition. CCA1 transcription factor up-regulates *terpene synthase* (*TPS)* by binding on CBS motif, 5’-AGATTTTT-3’ of promoter region to affect the circadian emission of floral scents in *Onc*. SB.

**Supplementary Information:**

The online version contains supplementary material available at 10.1186/s12870-022-03850-z.

## Background

Plants live in environments that oscillate with a period of approximately twenty-four hours. To adapt to the periodic environmental fluctuations such as light and temperature, they constantly monitor changes in the surrounding environment, and response to biological processes by a circadian rhythmic pattern. The circadian clock is an intrinsic and entrainable timekeeping mechanism, conferring plants to be able to buffer against both subtle and extreme changes in environment [1, 2]. Therefore, it contributes to timing multiple biological processes among various environmental factors. The role of circadian clock encompasses to almost every aspect of growth and developments. Generally, the detection on day-length changes is the most effective way to regulate gene expression. In addition to regulating daily activities, circadian clocks are also involved in the seasonal regulation of physiological function such as flowering, photoperiodism [3]. The circadian clock in plants is conducted by several circadian oscillator genes, which are clock components capable of generating the multiple negative regulatory feedback loops. Coordinated regulation of circadian oscillators and circadian-regulated output genes at various steps to establish and maintain circadian rhythms [4–6]. Recent progresses in genomic, biochemical and bioinformatics research have provided great understanding in the molecular architecture of the circadian clock in Arabidopsis [7, 8]. Most clock components possess transcriptional activity, and nearly 80% of transcriptomic genes in rice, poplar and Arabidopsis are regulated by internal circadian clock [9]. As a central circadian rhythm regulator, CCA1 occupied more than 1000 genomic regions identified in the seedling stage based on the CHIP sequencing data [10]. Among the targets of CCA1, although many of the target genes are repressed, some are activated, such as LHCB1.1 (CAB2) [11], this indicates the CCA1 is the master clock regulator containing broad function.

In the transcriptional level, CCA1 plays an important role in either repressor or activator in circadian rhythm, depending on which *cis*-element it has interacted with. As the further reports, CCA1 represses gene expressions when it interacts with EE (Evening Element; -AAATATCT-), and the peak of the gene expression in circadian rhythm highlights during 10–14 o’clock [12, 13]. In contrast, it works as an activator to induce the gene expressions of light-harvesting chlorophyll a/b protein: LHCB3.1, when it interacts with CBS element (-AAMAATCT-), and the peak of gene expression was at dawn [14]. It was also reported that when the EE element was point-mutated to the CBS element on the promoter of hydrogen peroxide catalase 3 (CAT3), the peak of circadian rhythm was shifted from 10–14 to 0–4 o’clock, suggesting that the circadian rhythm was affected by the *cis*-element which CCA1 interacted with [15].

Floral scent in plants is one of the most important floral characteristics to attract pollinators. The past studies on floral scent were generally assumed that floral scent is genetic adaption under the long-term pollinator-mediated natural selection, but recent evidence showed floral scent emissions are also influenced by environmental elements like light and temperature [16, 17]. To attract species-specific pollinators, floral scent emission usually oscillates with the circadian rhythm in parallel with insect activity [18–22]. For example, petunia emits fragrance mostly at night and less during the day, indicating a nocturnal rhythmic pattern [23, 24]. On the contrary, the floral emission of moth orchids is conducted with a diurnal rhythmic pattern [25]. It suggests that the rhythmic emission of floral scent is under the genetic adaption between plants and pollinators.

In this work, we used the *Oncidium* hybrid orchid, *Oncidium* Sharry Baby to study the molecular mechanism of its diurnal rhythmicity of floral scent emission. The synchronization between its terpenoid emission and gene expression of *terpene synthase* is an interesting biological process. Terpene synthase is one of the two key enzymes for synthesizing floral scent compounds, ocimene and linalool, in terpenoid biosynthetic pathway in *Onc*. SB. Ocimene and linalool are acyclic monoterpenes, produced by terpene synthase by different backbone rearrangement from the precursor geranyl diphosphate (GDP). Our survey on the promoter structure of *terpene synthase* disclosed the DNA motif of CIRCADIAN CLOCK ASSOCIATED 1 (CCA1) binding site (CBS). CCA1 is a key circadian clock component responsible for regulating the circadian rhythmic expression [3]. Our current study demonstrates that CCA1 appears to upregulate the expression of *terpene synthase*. Our finding elucidates the influence of the diel rhythm on scent emission in *Oncidium* Sharry Baby orchid.

## Results

### Floral scent is emitted by diel rhythmic pattern synchronizing with terpene synthase expression in *Oncidium* Sharry Baby

**Fig. 1:**
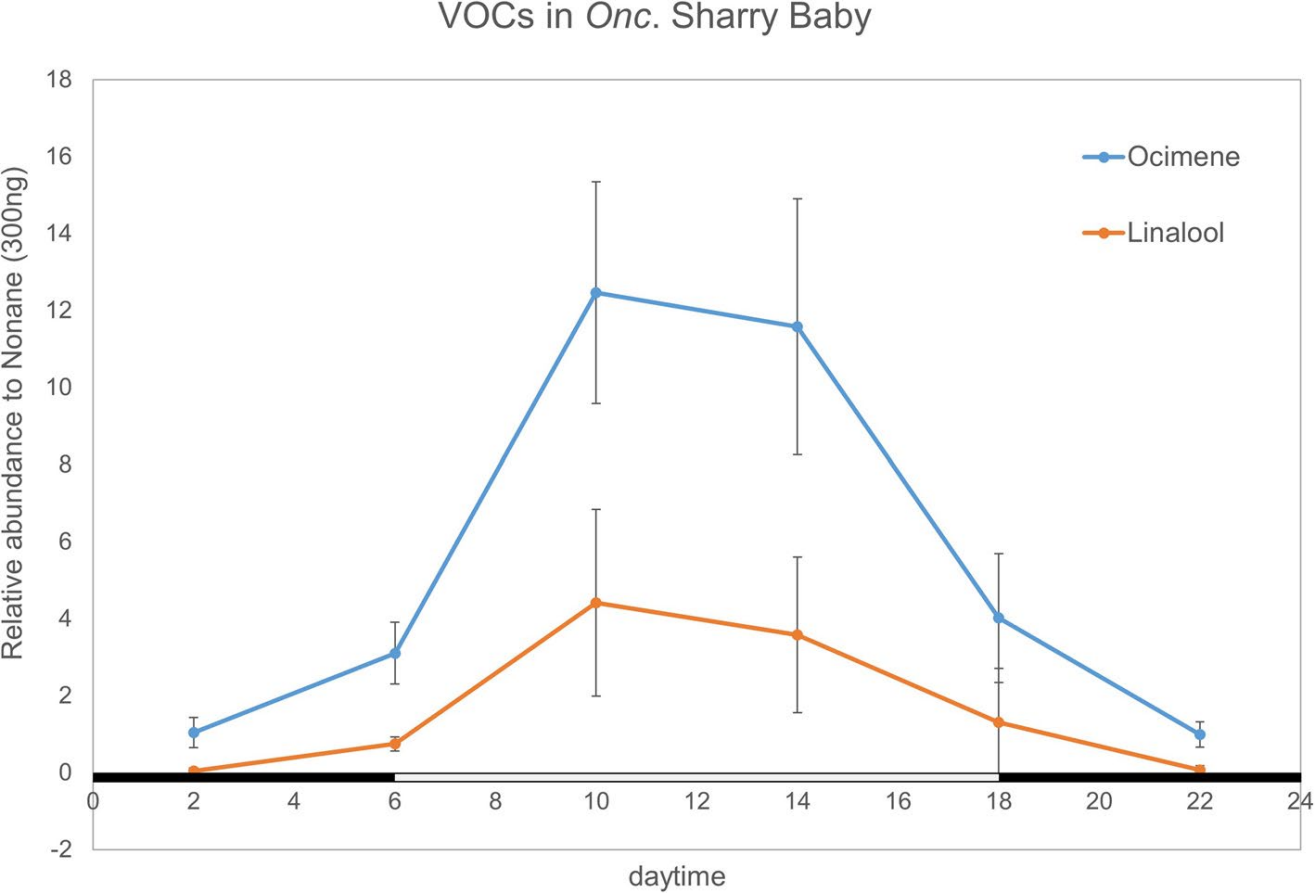
The emission patterns of two floral scent compounds, ocimene (blue line) and linalool (orange line) in *Oncidium* Sharry Baby on the whole day. The accumulation of floral scent emission rises from 6 o’clock, reaches the peak on 10 o’clock and starts decreasing from 14 o’clock until 22 o’clock. Data was analyzed by GC-Mass as described in Materials and Methods. Results represent mean ± SEM from three biological replicates

As our investigation, the floral volatile compounds were known as ocimene and linalool (Supplemental Fig. 1). Both of them were belong to monoterpenes, converted from geranyl diphosphate. In this work, we monitor its emission pattern on the whole day. Data revealed that the diel oscillation displayed a peak around 10 o’clock. The emission starts from 6 o’clock and decreases from 14 o’clock until 22 o’clock. The fragrant scents are dominant in the morning time, and vanish in the night time (Fig. 1). It suggests that the floral scent emission pattern is a diel control and regulated by oscillated genes.

### Diel rhythmic expression of terpene synthase is light-dependent, and abolished in constant light (LL) and constant dark (DD) environment

**Fig. 2:**
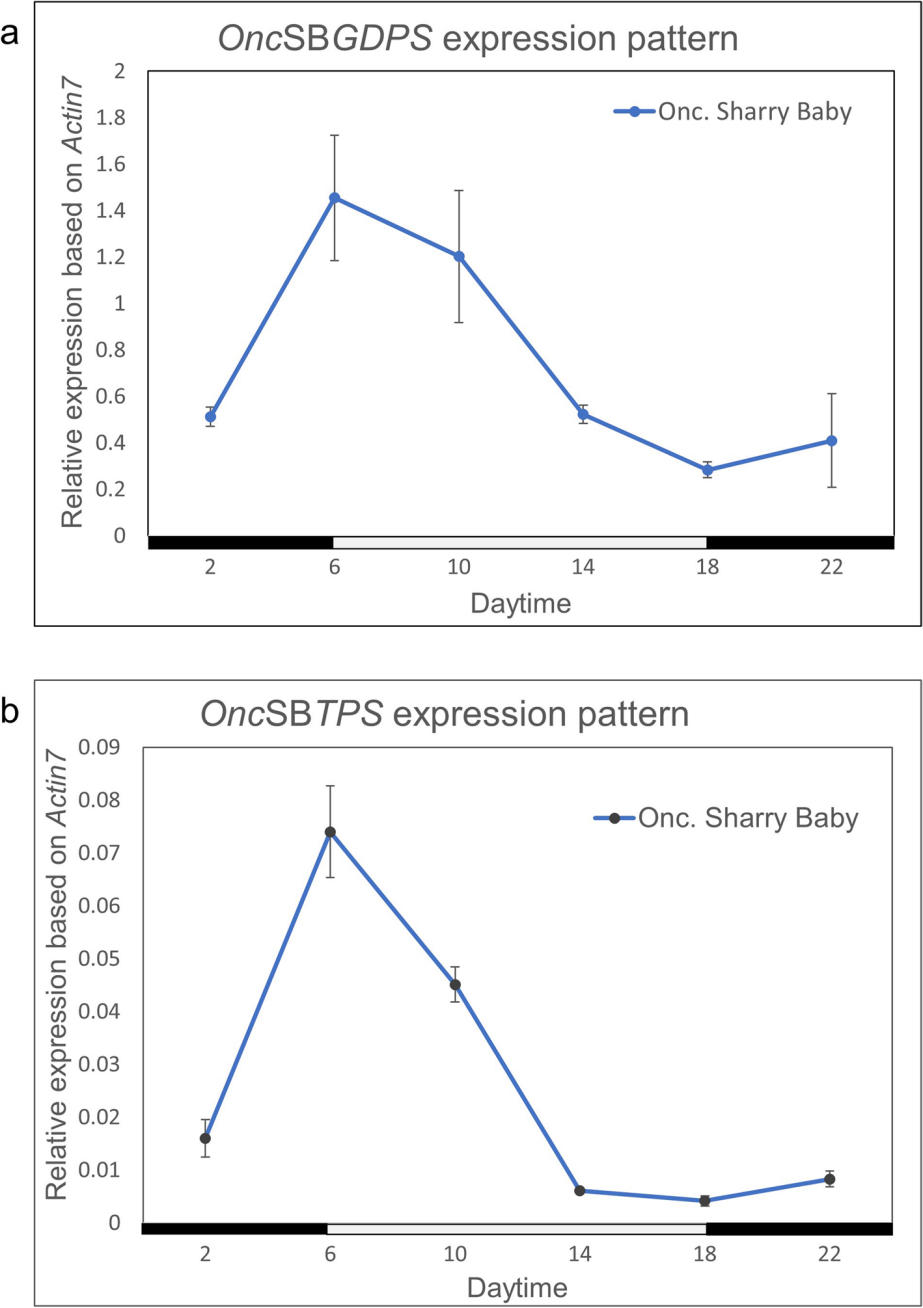
Gene expression levels of *geranyl diphosphate synthase* (**a**), and *terpene synthase* (**b**) in *Oncidium* Sharry Baby. Data revealed that *GDPS* is highly expressed whole the day, while *TPS* is expressed relatively less with an enormous peak at 10 o’clock. Results represent mean ± SEM from three biological replicates

**Fig. 3:**
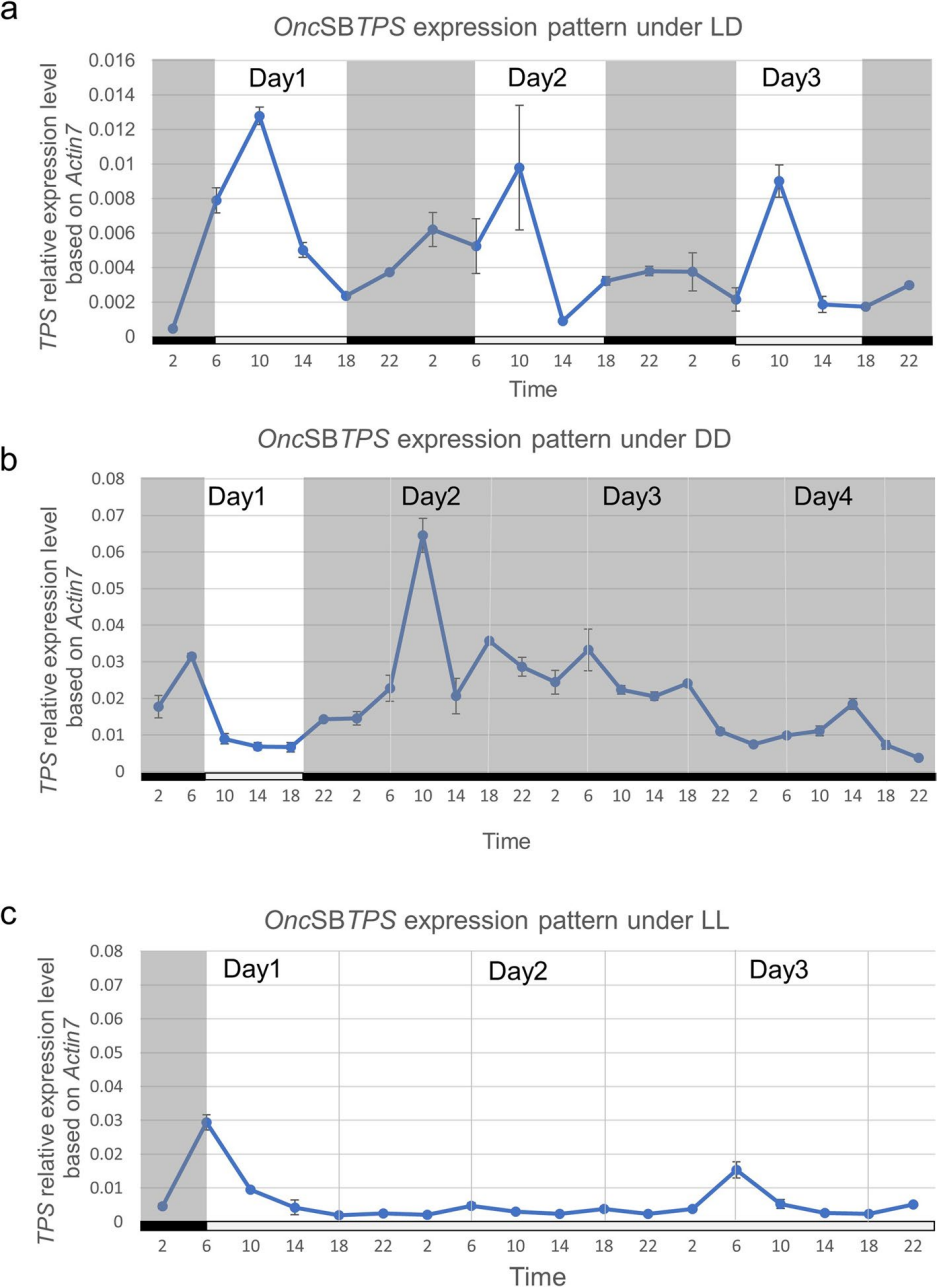
Assay on the diel rhythmic expression pattern of *Onc*SB*TPS* under different light condition*.* Results represent mean ± SEM from three biological replicates. **a** The gene expression patterns of *Onc*SB*TPS* under 12 h light/12 h dark (LD). The peak of gene expression level daily was on 10 o’clock during the 72 h period. **b** The gene expression patterns of *Onc*SB*TPS* under constant dark (DD) during 94 h period. When in constant dark, the peak of gene expression levels and the rhythmic periods were shortened, and abolished gradually. **c** The gene expression patterns of *Onc*SB*TPS* in constant light (LL) during 72-h period. the rhythmic expression pattern was abolished in the constant light

Both geranyl diphosphate synthase (GDPS) (gene accession number: MH171283.1) and terpene synthase (TPS) (gene accession number: MH171282.1) constitute the two-step enzymes of the biosynthetic pathway to produce monoterpene compounds. The GC–MS data suggested that the final product was linalool and ocimene (supplemental Fig. 2). However, because the reaction condition was not similar with the nature circumstance, the ratio of both terpene compounds was also different. While we check the expression pattern of both *GDPS* and *TPS*, *GDPS* is highly expressed whole the day with a peak at 2–10 o'clock (0.5〜1.5 relative expression level; Fig. 2a), while *TPS* is much less expressed (0.01〜0.07 relative expression level), but it shows an enormous peak around 10:00 o’clock in parallel with the emission pattern of floral scent (Fig. 2b). In conclusion, TPS is a bottle-neck enzyme in the floral scent biosynthesis. However, the *TPS* expression pattern is closely coincident with the emission pattern of ocimene/linalool, suggesting *TPS* could determine the diel rhythmicity of floral scent biosynthesis (Fig. 2b). Therefore, the investigation on expression patterns to understand the diel rhythm is necessary. Furthermore, we conducted a 72-h time course experiment to monitor the alternation of expression levels of *TPS* under growth conditions of 12L/12D, DD (constant dark) and LL (constant light) respectively by using a 2-week-old floral plants. While the diel experiment is regularly operated during the 12 h, L/12 h, D condition, data revealed that *TPS* gene expression is following the diel rhythm during 72-h experiment. The diel rhythmic pattern of gene expression levels maintained at similar intensities and oscillations, when light and dark signal was switched regularly (Fig. 3a). In contrast, the diel oscillation is abolished in both DD and LL growth condition (Fig. 3b, c). It suggests that the light–dark signal, rather than the circadian clock, regulates the *TPS* gene expression.

### *TPS* promoter region containing CCA1-binding site (CBS), and circadian rhythmic expression of CCAl directly regulates *TPS* transcription

**Fig. 4:**
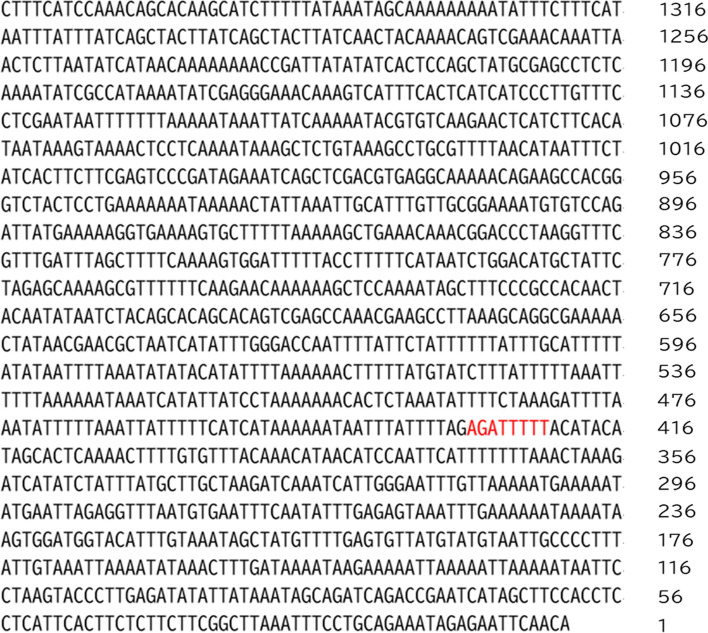
The primary structure analysis showing *cis*-acting elements in *TPS* promoter region (~ 1375 bp length). The putative CCA1 binding site (CBS), -AGATTTTT- located at -423 ~ -430 bp of *TPS* promoter was marked by red color

**Fig. 5:**
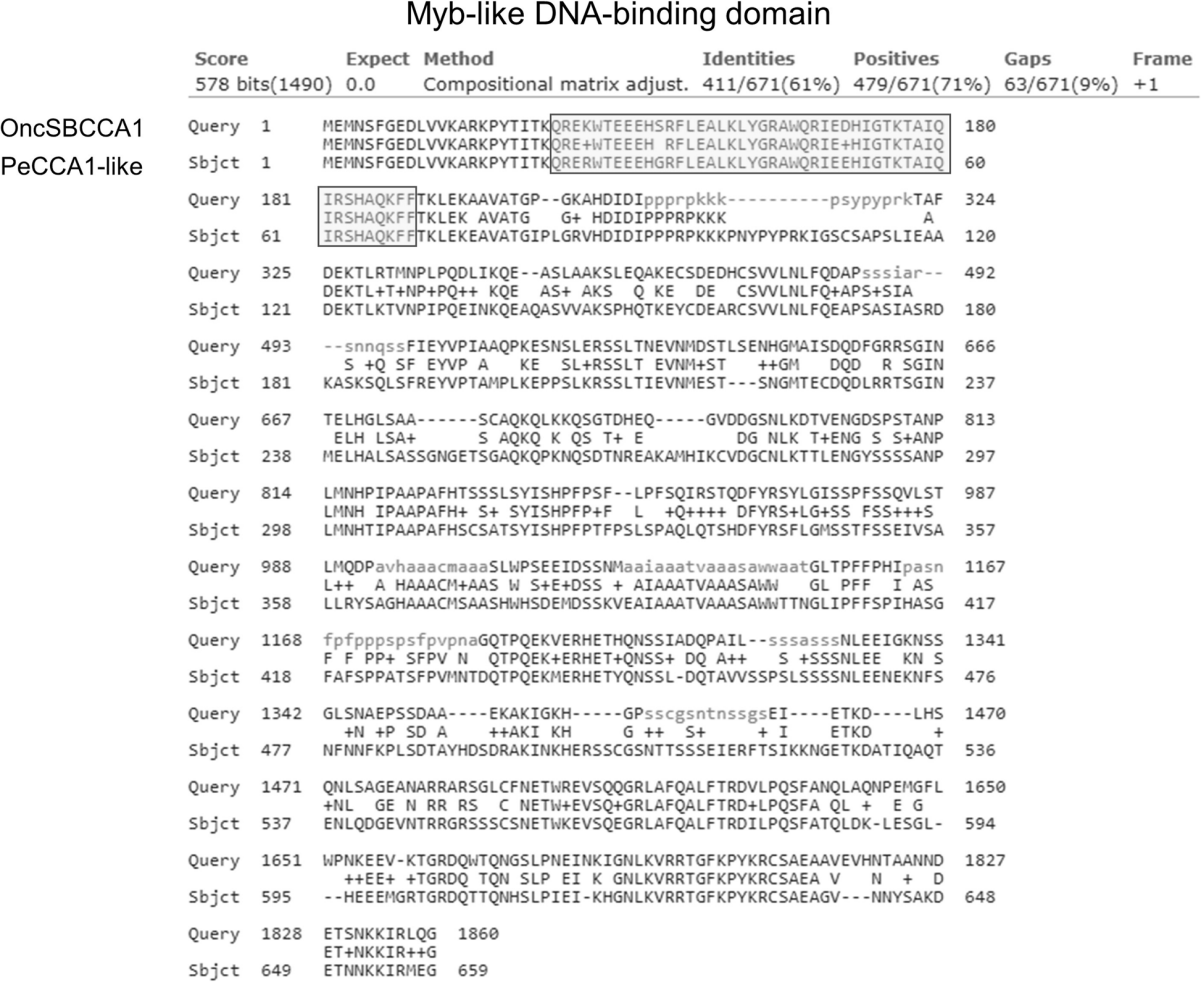
Sequence comparison between *OncSB*CCA1 and *Pe*CCA1-like amino acid sequences. Although they only shared 61% identity, there is a high conservation in the Myb-like binding domain (marked by frame), suggesting that CCA1 also have Myb-like function. *Onc*SB: *Oncidium* Sharry Baby; Pe: *Phalaenopsis equestries*

**Fig. 6:**
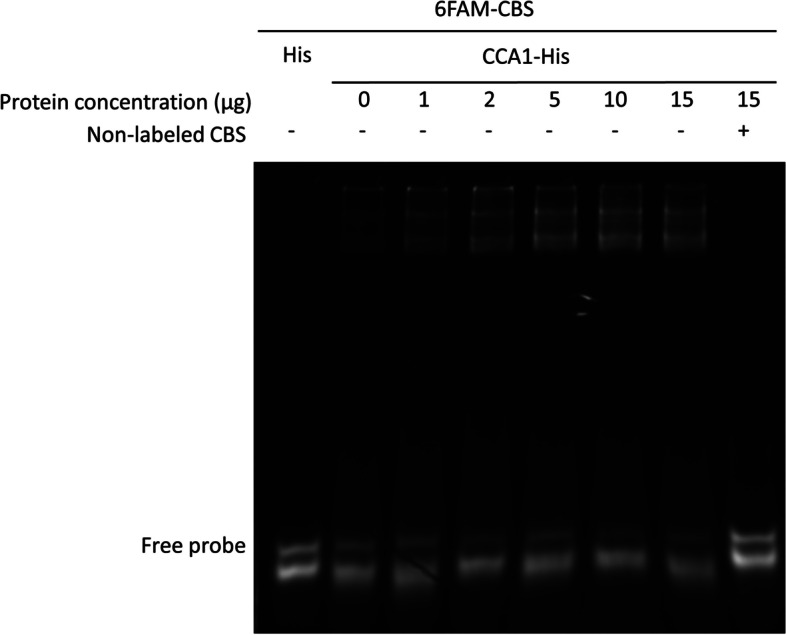
EMSA profile showing the interaction of CBS DNA motif and CCA1 protein. The CBS double- strand DNA (24-mer) was initially labelled with 6-FAM fluorescence dye as probe. The labelled probe sample (0.7 μL) each was reacted with various concentration of CCA1 protein. When 50 × unlabeled probe was added, fluorescence signal of CCA1-probe interaction was disappeared. When the concentration of CCA1 protein (µg) was gradually increased, fluorescence intensity of total free probes decreased, suggesting the presence of affinity between CCA1 and CBS element

We further isolate *TPS* promoter region (~ 1375 bp length) and analyze the *cis*-acting elements of promoter structure by accessing PlantCARE website (http://bioinformatics.psb.urgent.be/webtools/plantcare/html/). Obviously, there is a putative CCA1 (Circadian Clock Associated 1) binding site (CBS), -AGATTTTT- located at -423 ~ -430 bp in *TPS* promoter region (Fig. 4). In order to study the regulatory function of CCA1 on *TPS* transcription. A full *Onc*SB*CCA1* cDNA sequence was further cloned based on the *CCA1* contig sequences existing in the available transcriptomic database (Gene Bank accession number GIQW00000000). cDNA sequence containing 1,869 bp ORF and with the deduced protein molecular weight of 67.9 kDa was completed. Its cDNA sequence showed a higher homology to *Phalaenopsis equestries* (moth orchid) (Fig. 5). Notably, only one conspicuous MYB motif is present in each cDNA sequence, suggesting it is a single MYB- domain type of *Onc*SBCCA1 transcription factor (Fig. 5). Furthermore, the cDNA CDS fragment was subcloned in pET28a expression vector and was transformed to *E. coli* BL21(DE3) for recombinant protein production. The recombinant protein was purified through High Affinity Ni-Charged Resin column following the general protocol. Analysis on SDS-PAGE profile displayed a 68.74 kDa His-tag fused protein band (data not shown), indicating the overexpressed recombinant protein is coincident with the predicted molecular weight of CCA1. Furthermore, Electrophoresis mobility shift assay (EMSA) was performed to identify the binding activity between CCA1 protein and CBS *cis*-acting motif of *TPS* promoter region. The DNA probe, was synthesize as -AAAAATCT-, and added to react with *Onc*SBCCA1 recombinant protein as described in Materials and Methods. The significant fluorescence signals shown on 10% native PAGE data clearly appear at the top of gel, and the signal intensity increases in accordance with the gradually increasing concentration of CCA1(Fig. 6, Supplemental Fig. 3). This data confirmed the specific affinity between CCA1 protein factor and CBS *cis*-acting sequence. EMSA data demonstrate the binding activity of CCA1 on *TPS* promoter. Moreover, it strongly suggests that CCA1 can directly regulate *TPS* gene expression.

**Fig. 7:**
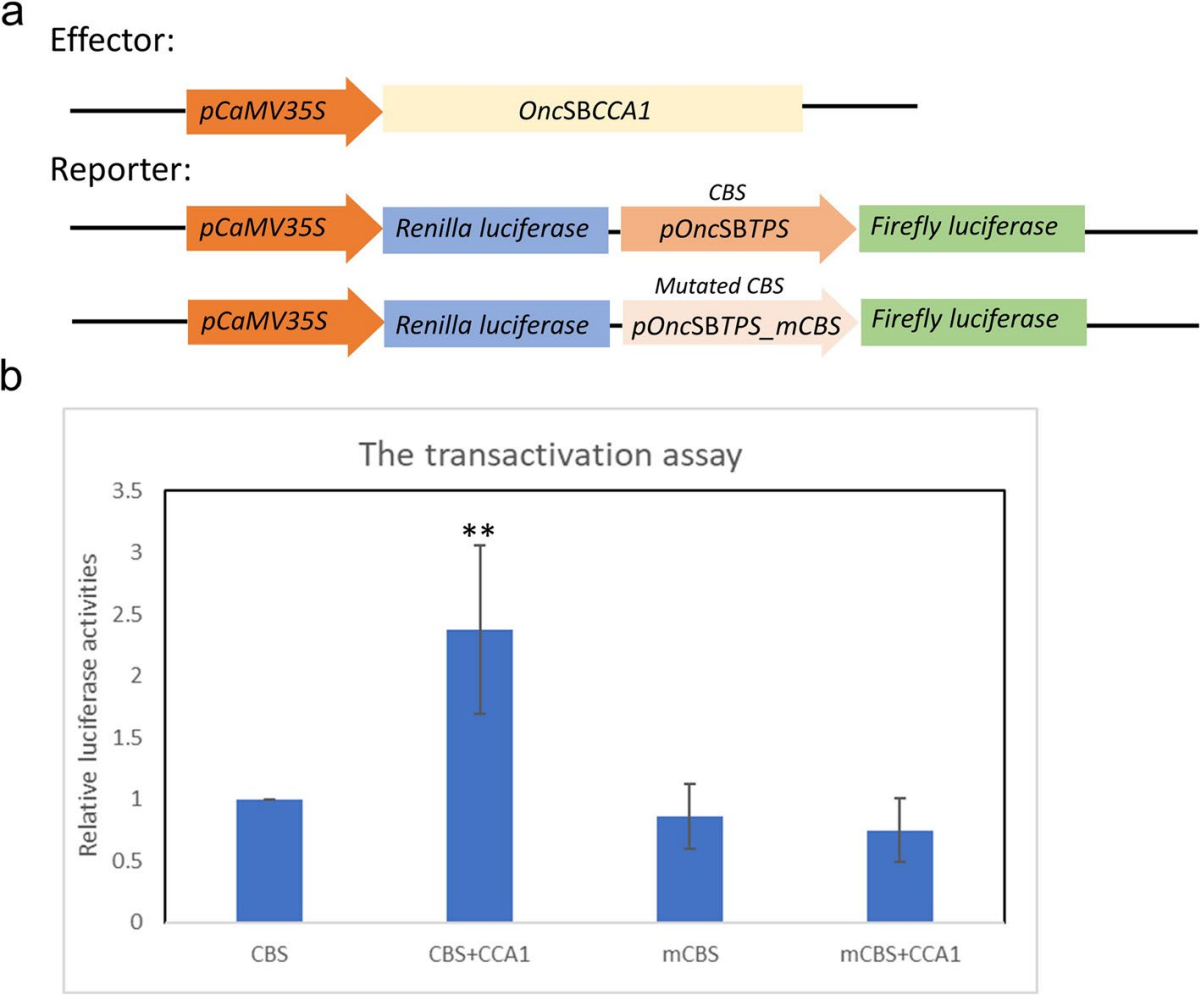
Transactivation assays showing CCA1 regulation on *TPS* promoter activity by binding on CBS motif **a** The schematic model of effector and reporter constructs. *Onc*SB*CCA1* was driven by *pCaMV35S* promoter and *pOnc*SB*TPS* was used to drive Firefly Luciferase. As a negative control, CBS (-AGATTTTT-) on the *pOnc*SB*TPS_mCBS* was replaced as -GGGTTTTT-, in order to prevent the interactions with *Onc*SBCCA1 transcription factor. **b** The promoter activities assay with or without *Onc*SBCCA1 transcription factor. Firefly Luciferase activities were detected to represent the interactions between CCA1 and CBS. The data suggested that *Onc*SBCCA1 upregulates the gene which was driven by the promoter containing CBS (*n* = 7, *P* < 0.05: *, *P* < 0.01: **)

To further demonstrate the regulatory interactions between CCA1 and CBS, transactivation assay was performed. The result showed that when CCA1 interacted with CBS, the fluorescence accumulation was significantly higher than those one in which CCA1 interacted with mutated CBS, suggesting that CCA1 upregulated *TPS* expression by binding on CBS motif of promoter (Fig. 7).

### Circadian rhythmicity of *CCA1* is responsible for regulating *TPS* transcription

**Fig. 8:**
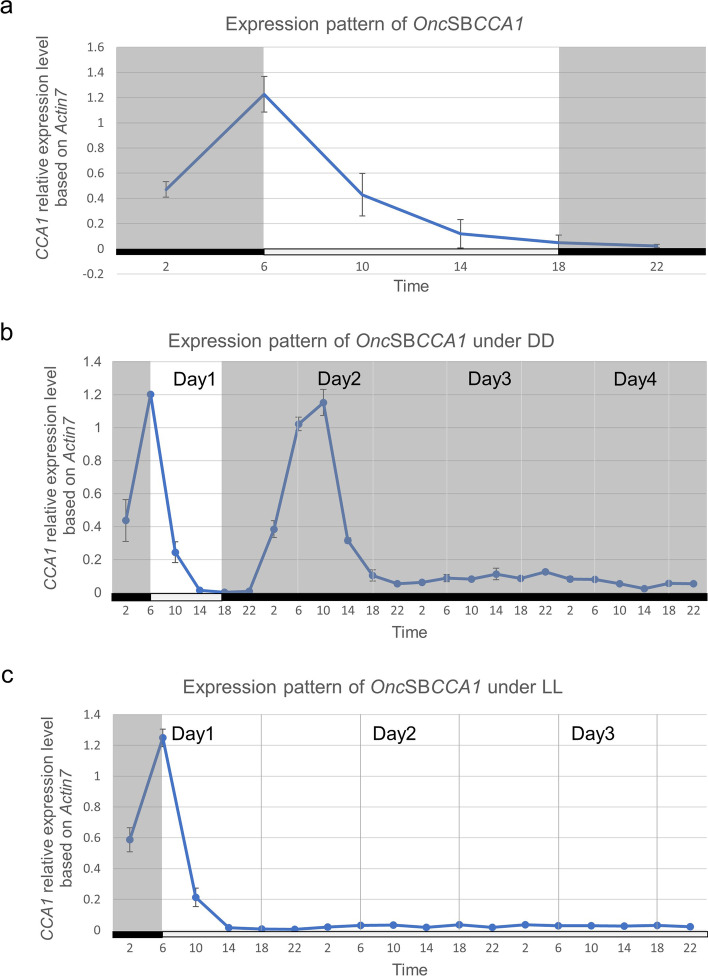
Gene expression pattern of *CCA1* in flower organ on the whole day (**a**), under constant light 12L/12L for 72-h course (**b**), and constant dark 12D/12D for 72-h course (**c**). The expression level of *CCA1* exhibited a diel rhythm 12L/12D for 72 h course, indicating *CCA1* expression pattern is in parallel with expression pattern of *TPS* and emission pattern of floral scents. Results represent mean ± SEM from three biological replicates

*CCA1* has been known as a core oscillator gene in circadian network [10]. Although our data has confirmed the binding activity on CBS morning element of *Onc*SB*TPS* (Fig. 6, Supplemental Fig. 3), the expression pattern of *Onc*SB*CCA1* in *Onc*. Sharry Baby is still unclear. To verify the circadian expression of *Onc*SB*TPS* is coordinately regulated by CCA1, the expression pattern of *Onc*SB*CCA1* in flower organ was monitored by qRT-PCR. As shown in Fig. 8a, the *CCA1* expression is active from 6:00 to 10:00 o’clock. It indicated a diel expression type, and in coincidence with *Onc*SB*TPS* expression pattern (Fig. 2b). Further, we conducted a 72-h time-course experiment to monitor the expression by using 2-week-old floral plants grown under 12L/12L (Fig. 8b) and 12D/12D respectively (Fig. 8c). The expression level of *CCA1* clearly exhibited a rhythm, indicating *CCA*1 expression pattern is in accordance with expression pattern of *Onc*SB*TPS* and emission pattern of the floral scents.

## Discussion

To ensure the fine control of metabolic processes associated with the physiological functions, plants have developed an endogenous system for a precise measurement of photoperiod, represented by circadian rhythms, synchronized with the prevailing environmental conditions. The system is the regulatory mechanism of gene expression. For example, the temporal regulation of plant scent emission is correlated with the expression pattern of biosynthetic genes in the metabolic pathway of scent production [26–28]. The scent emission is closely tied with the oscillation of expression pattern of metabolic genes. In the scent biology, several model plants, such as rose, snapdragon, tobacco, and petunia have been comprehensively studied for the oscillatory emission patterns. In this regard, *Petunia* hybrida has been perhaps the most complete model in scent emission [29]. The analysis of gene expression within the floral volatiles benzenoid/ phenylpropanoid (FVBP) pathway provided key insights into the mechanism of scent regulation. Thus far, most studies of the regulatory mechanism of oscillatory emission of floral volatiles are focused on FVBP pathway. FVBP pathway is composed of a series of enzymes. For example, the oscillation of methyl benzoate compound is closely correlated with the expression patterns of the biosynthetic enzymes- *BSMT1* and *BSMT2* (Salicylate/benzoate carboxyl methyltransferase 1 and 2). *PAL* (*phenylalanine lyase*) mRNA expression pattern also oscillates in a similar pattern to that of benzoic acid synthesis in diurnal conditions [30]. Recently, two R2R3-type MYB transcription factors, *ODORANT1* (*ODO1*) and *EMISSION BENZENOIDS II* (*EOBII*) were identified as regulatory components of floral scent metabolism. Both are involved in FVBP synthesis [31]. Up-regulation and down-regulation of their transcription increases and decreases the transcripts of many key enzymes in the FVBP pathway and subjective to affect scent production. Most recently, it revealed the identification of putative binding sites of clock gene, i.e., *LHY* (*LATE ELONGATED HYPOCOTYL*) in the promoter of *ODO1* of *P. hybrida* [32]. When *PhLHY* was over-expressed, the floral emission of hybrida was almost lost, and many genes involved in FVBP pathway, including *ODO1*, *EPSPS* (*enolpyruvylshikimate 3-phosphate synthase*), *CM1* (*chorismate mutase 1*), *ADT* (*arogenate dehydratase*), and *PAL*, were down-regulated [15]. However, when *PhLHY* was mutated, the peak of floral scent emission and gene expression levels in FVBP pathway were move up from dusk to afternoon. As its homologs in other plants, *PhLHY* peaks around dawn [26]. Further investigation reported it can bind to other genes in the FVBP pathway and controls the expression phase of these genes. These researches delivered the information that temporal expression of scent appears to be primarily regulated through manipulation of the timing of transcriptional regulators in the biosynthetic pathway [16]. In recent years, MYB transcription factors were reported to regulate the production of terpenoids [7].

Our current study firstly found that the floral scent emitted from *Onc*. Sharry Baby closely related with the expression of *terpene synthase*. Its relative less expression level influences the scent quantity. The circadian expression is controlled by the promoter structure. Analysis on promoter structure of *Onc*SB*TPS* disclosed a putative CBS (CCA1 binding site), -AGATTTTT-, located at -423〜-430 bp of *TPS* promoter (Fig. 4). The EMSA assay identified that *Onc*SBCCA1 transcriptional factor is able to bind on CBS sequence of *TPS* promoter (Fig. 6). Transactivation assay confirmed the CCA1 regulation on the *TPS* promoter by binding CBS motif (Fig. 7). Similar to the emission pattern of floral scent, *TPS* and *CCA1* are expressed at peak of 6:00〜10:00 o’clock in the morning, demonstrating the circadian clock function in scent emission. Interestingly, circadian oscillation of *Onc*SB*TPS* and *Onc*SB*CCA1* expression showed distinct patterns in light- dependent manners. When *Onc.* SB orchid plants were kept in continuous light (LL) and continuous dark, robust oscillation of *Onc*SB*TPS* and *Onc*SB*CCA1* abolished in the first day (Fig. 3). The results indicated that the proper light/dark cycle is the critical condition for robust circadian oscillation of *Onc*SB*TPS* and *Onc*SB*CCA1* in *Onc.* SB. Evidence suggested that both light and internal clock mutually interact in the rhythmic expression of clock genes.

## Conclusion

In the present work, we address the genetic mechanism how the circadian clock controls the floral volatiles emission in *Onc.* SB orchid. *CCA1* plays role of the core oscillator, directly regulating *terpene synthase* transcription, by which volatiles of ocimene and linalool were synthesized timely in floral tissues. The robust diel rhythmicity is synchronizing among core oscillator, rhythm-regulated gene expression levels, and scent emission period. The rhythmic function occurs in terpenoid metabolic pathway in *Oncidium* orchids was first demonstrated.

## Materials and methods

### Plant materials and growth condition

*Oncidium* orchid plants were purchased from Xu-Tung nursery Co. Thirty plants as early-blooming stage two weeks prior to experiments were grown in greenhouse at 30 ℃/25 ℃ (14 h light/10 h dark). When we investigate the circadian rhythm of gene expression levels and VOC emission of *Oncidium* orchids, plants were grown in 25 ℃, constant light, or constant dark, or 12 h light/ 12 h dark, which switches at 6 and 18 o’clock, environment conditions. The light intensity in this study was 250–300 μmolm^−2^ s^−1^. Three independent plants with full blooming were used to measure volatile emission and circadian rhythmic assays. A single flower from an independent orchid plant was used in each sample for the study of timing emission. Total three flowers were used to measure and evaluated on average.

### Floral volatile collection and GC–MS analysis

The solid-phase micro-extraction (SPME) technique coupled with GC–MS system was used to collect and analyze floral volatile compounds, following the method described by Lin et al. [25]. The whole floret organs were cut at 2:00, 6:00, 10:00, 14:00, 18:00 and 22:00 o’clock separately and immediately sealed in 20 ml headspace sample vials with 50 μm SPME fiber (Sigma-Aldrich, MO, USA) following injection of 100 ng of an internal standard *n*-nonane. The volatiles absorbed by the SPME fiber was directly desorbed for 5 min in the GC inlet. The GC–MS system is a Trace GC Ultra Gas Chromatograph and a Polaris Q mass spectrometer (Thermo Fisher Scientific, USA). The GC column was a 30 m × 0.25 mm × 0.25 μm column (DB-5 ms, Agilent Technologies). The GC oven temperature process was at 60 °C initially, then rose up to 220 °C at 4 °C/min and stay in 220 °C for 2 min. Afterwards, the temperature ramped to 250 °C and held for 3 min with the heating speed 20 °C/min. The temperature of inlet is 250 °C. The carrier gas is helium (99.9995%) and the flow speed was 1 mL/min. The split ratio is 10:1. The temperature of transferline and ion source of MS are 250 °C and 230 °C, respectively. The ionization energy is 70 eV and the ion scan range of MS is 50 – 400 amu. The identification of the component of floral scent is using the Kovats index, MS spectra from NIST. VOC data was collected from three floral individuals each from one of three different blooming orchid plants and calculated by average.

### Gene expression analysis

Total RNA of floral tissues was extracted by using the pine tree method [33]. The real-time quantification PCR (ABI 7500, Thermo Fisher Scientific, MA, USA) was performed to assay gene expression profile following the manufacturer’s protocol (The KAPA SYBR qPCR kit, Wilmington, USA). The gene expression patterns of *Onc*SB*TPS* (*terpene synthase*) were determined by the primer pair of 5’-CTCTGGATGTGCCTTTGGTCAGAAG-3’ and 5’-AGCTCTTCCACAGTCCCATAATTATCATA-3’. For *CCA1*, the primer pair was: 5’CAAGCTTGTCGTGAGATATTCTCATTTGC-3’ and 5’-TCAAACTTTTCCTTGCAGACGAATTTTC-3’. The internal standard used in this study was actin 7 cloned from *Oncidium* SB.

### Electrophoresis mobility shift assay

Two primers, which were designed based on the nucleotide sequence of candidate genes in *Oncidium* SB transcriptome (Gene Bank accession number GIQW00000000), Contig TR3792_c0_g1_i1 was employed to amplify the *CCA1* cDNAs of *Onc*. SB. The forward and reverse primers were as following: 5’-ATGGAGATGAACTCTTTTGGAGAAGATC-3’ and 5’-TCAAACTTTTCCTTGCAGACGAATT-3’, respectively. The full-length *CCA1* CDS was cloned into the pET28a vector, transformed and expressed in *E. coli* BL21(DE3). Transformed *E. coli* containing the pET28a-*CCA1* vector was grown in 50 mL of LB broth at 37 ℃ overnight. After cell culture growing to reach OD_595_ 〜 0.5, 100 μL IPTG (0.1 M) was added to induce *CCA1* expression for 4 h. Expressed proteins were harvested and purified by using a His column (Genscript, New Jersey, USA) following the manufacturer’s instructions. EMSA performance was conducted as described by Chen et al*.* [34]. Experiment was carried out in photophobic condition. At the beginning, the 6-FAM fluorescence dye was ligated to a 24-nucleotide of the double-strand CBS DNA, -TATTTTAGAGATTTTTACATACAT-, by T4 ligase to produce fluorescent probe with the following ingredients: 0.7 μL probe, 2μL 5X binding buffer (50 mM Tris–HCl, 750 mM KCl, 2.5 mM EDTA, 62.5% glycerol), 0.1μL 100X BSA, different dosage (0, 1, 2, 5, 10, 15 μg) of CCA1 recombinant protein, and adding H_2_O to final volume of 10μL. 50 × double-strand CBS DNA without ligating 6-FAM was used as competition DNA. Reaction mixtures was stood for 30 min before loaded to electrophoretic gel. Native gel was prepared by 3.5 mL 10X TAE, 4.5 mL 40% acrylamide, 8 mL H_2_O, 8μL TEMED. The EMSA gel electrophoresis was performed in 0.5X TAE buffer and powered by150V. The fluorescence was detected by LAS3000 (Fujifilm, Tokyo, Japan) by using FAM mode, and 30 s for exposure time.

### Transactivation assay

The technology of transactivation assay was following the protocol of Sherf et al., 1996 [35]. Arabidopsis protoplast was prepared in which plants were grown in 25℃short day growth chamber for 4 weeks. The cut-off leaves were soaked in 5 mL enzyme solution (cellulose R10 1.5%, Macerozyme R-100 0.4% (Yakult, Tokyo, Japan), 20 mM MES (pH5.7), 0.4 M mannitol, 20 mM KCl, and 10 mM CaCl_2_·2H_2_O) in the dark room for 5 h. the reaction product was filtered and centrifuged for 80xg, 1 min, and then discarded the supernatant. The pellet was redissolved in 1 mL W5 solution (154 mM NaCl, 125 mM CaCl_2_·2H_2_O, 5 mM KCl, 2 mM MES (pH5.7), 5 mM glucose) under 4℃.

Partial promoters of *pOnc*SB*TPS*(-1 ~ -441 bp) was cloned from *Onc*. SB genome. *pOnc*SB*TPS*_mCBS, in which CBS was mutated from -AGATTTTT- to -GGGTTTTT-. Both were cloned into vector pGreenII0800-Luc as a reporter construct. In the reporter construct, *pCaMV::Renilla luciferase* was an internal standard. The full length *CCA1* was driven by CaMV 35S promoter in effector vector. Two vectors were co-transfected into protoplasts. The Dual-Luciferase Reporter Assay System (Promega, WI, USA) was applied to perform the Luciferase Assay. The firefly luciferase activities were normalized by the *Renilla luciferase* activities.

### Statistics

To investigate the Significant difference in the transactivation assay, one way ANOVA was applied. The program to calculate the statistics was Excel 2019 (Microsoft corp., Washington, U.S.A), and the Add-in was Real-statistics (https://www.real-statistics.com/). The Post Hoc test was Tukey HSD Test.

## Supplementary Information

**Additional file 1: Supplemental Fig. 1.** The GC-MS spectrum of floral scent from *Oncidium *SB flowers in different times. The volatile terpenes, ocimene and linalool display the peaks at RT=11.31 and 12.91 respectively. The profiles decipher floral scents are diurnally emitted highly at 10:00 o’clock and lower at 18:00 o’clock. The GC spectrum showed that there is a circadian rhythm of floral scent emissions. **Supplemental Fig. 2.** The enzymatic activities assay of TPS. TPS recombinant protein was purified, then fed with the precursor GDP, and reacted at 37℃ for 4hrs. The GC-MS data suggested that the final product was linalool and ocimene. The SPME and GC-MS analysis conditions was described in text. **Supplemental Fig. 3.** The uncropped image of Fig. 6 was attached. In order to ensure the compliance with the digital image and integrity policies, the uncropped image of Fig. 6 was attached. At two lanes on the right, we increased the concentration of CCA1 when the competitor was added. As the result showed, the fluorescence intensities of free probe decreased. To focus on the interaction between CCA1 and CBS, Figure 6 didn’t show these two lanes.

## Data Availability

The datasets used and/or analyzed during the current study are available from the corresponding author on request.

## References

[1] Bell-Pedersen D, Cassone VM, Earnest DJ, Golden SS, Hardin PE, Thomas TL, Zoran MJ (2005). Circadian rhythms from multiple oscillators: Lessons from diverse organisms. Nat Rev Genet.

[2] Yerushalmi S, Green RM (2009). Evidence for the adaptive significance of circadian rhythms. Ecol Lett.

[3] Nagel DH, Kay SA (2012). Complexity in the wiring and regulation of plant circadian networks. Curr Biol.

[4] Pruneda-Paz JL (2009). A functional genomics approach reveals CHE as a component of the Arabidopsis circadian clock (vol 325, pg 1481, 2009). Science.

[5] Yakir E, Hassidim M, Melamed-Book N, Hilman D, Kron I, Green RM (2011). Cell autonomous and cell-type specific circadian rhythms in Arabidopsis. Plant J.

[6] Srivastava D, Shamim M, Kumar M, Mishra A, Maurya R, Sharma D, Pandey P, Singh KN (2019). Role of circadian rhythm in plant system: An update from development to stress response. Environ Exp Bot.

[7] Shim JS, Imaizumi T (2015). Circadian Clock and Photoperiodic Response in Arabidopsis: From Seasonal Flowering to Redox Homeostasis. Biochemistry-Us.

[8] Millar AJ (2016). The Intracellular Dynamics of Circadian Clocks Reach for the Light of Ecology and Evolution. Annu Rev Plant Biol.

[9] Filichkin SA, Breton G, Priest HD, Dharmawardhana P, Jaiswal P, Fox SE, Michael TP, Chory J, Kay SA, Mockler TC (2011). Global Profiling of Rice and Poplar Transcriptomes Highlights Key Conserved Circadian-Controlled Pathways and *cis*-Regulatory Modules. Plos One.

[10] Nagel DH, Doherty CJ, Pruneda-Paz JL, Schmitz RJ, Ecker JR, Kay SA (2015). Genome-wide identification of CCA1 targets uncovers an expanded clock network in Arabidopsis. P Natl Acad Sci USA.

[11] Wang ZY, Tobin EM (1998). Constitutive expression of the CIRCADIAN CLOCK ASSOCIATED 1 (CCA1) gene disrupts circadian rhythms and suppresses its own expression. Cell.

[12] Harmer SL, Kay SA (2005). Positive and negative factors confer phase-specific circadian regulation of transcription in Arabidopsis. Plant Cell.

[13] Harmer SL (2009). The Circadian System in Higher Plants. Annu Rev Plant Biol.

[14] Wang ZY, Kenigsbuch D, Sun L, Harel E, Ong MS, Tobin EM (1997). A Myb-related transcription factor is involved in the phytochrome regulation of an Arabidopsis Lhcb gene. Plant Cell.

[15] Michael TP, McClung CR (2002). Phase-specific circadian clock regulatory elements in Arabidopsis. Plant Physiol.

[16] Sagae M, Oyama-Okubo N, Ando T, Marchesi E, Nakayama M (2008). Effect of temperature on the floral scent emission and endogenous volatile profile of Petunia axillaris. Biosci Biotech Bioch.

[17] Hu ZH, Zhang HX, Leng PS, Zhao J, Wang WH, Wang SD. The emission of floral scent from Lilium “siberia” in response to light intensity and temperature. Acta Physiol Plant. 2013;35(5):1691–700.

[18] Dudareva N, Martin D, Kish CM, Kolosova N, Gorenstein N, Faldt J, Miller B, Bohlmann J (2003). (E)-beta-ocimene and myrcene synthase genes of floral scent biosynthesis in snapdragon: Function and expression of three terpene synthase genes of a new terpene synthase subfamily. Plant Cell.

[19] Dudareva N, Negre F, Nagegowda DA, Orlova I (2006). Plant volatiles: Recent advances and future perspectives. Crit Rev Plant Sci.

[20] Dudareva N, Klempien A, Muhlemann JK, Kaplan I (2013). Biosynthesis, function and metabolic engineering of plant volatile organic compounds. New Phytol.

[21] van der Niet T, Johnson SD (2012). Phylogenetic evidence for pollinator-driven diversification of angiosperms. Trends Ecol Evol.

[22] Fenske MP, Imaizumi T (2016). Circadian Rhythms in Floral Scent Emission. Front Plant Sci.

[23] Simkin AJ, Underwood BA, Auldridge M, Loucas HM, Shibuya K, Schmelz E, Clark DG, Klee HJ (2004). Circadian regulation of the PhCCD1 carotenoid cleavage dioxygenase controls emission of beta-ionone, a fragrance volatile of petunia flowers. Plant Physiol.

[24] Underwood BA, Tieman DM, Shibuya K, Dexter RJ, Loucas HM, Simkin AJ, Sims CA, Schmelz EA, Klee HJ, Clark DG (2005). Ethylene-regulated floral volatile synthesis in petunia corollas. Plant Physiol.

[25] Lin CY, Chen YH, Chang TC, Chen YJ, Cheng SS, Chang ST (2013). Characteristic Aroma-Active Compounds of Floral Scent in Situ from Barringtonia racemosa and Their Dynamic Emission Rates. J Agr Food Chem.

[26] Fenske MP, Hazelton KDH, Hempton AK, Shim JS, Yamamoto BM, Riffell JA, Imaizumi T (2015). Circadian clock gene LATE ELONGATED HYPOCOTYL directly regulates the timing of floral scent emission in Petunia. P Natl Acad Sci USA.

[27] Fenske MP, Nguyen LP, Horn EK, Riffell JA, Imaizumi T (2018). Circadian clocks of both plants and pollinators influence flower seeking behavior of the pollinator hawkmoth Manduca sexta. Sci Rep-Uk.

[28] Bloch G, Bar-Shai N, Cytter Y, Green R (2017). Time is honey: circadian clocks of bees and flowers and how their interactions may influence ecological communities. Philos T R Soc B.

[29] Cna'ani A, Muhlemann JK, Ravid J, Masci T, Klempien A, Nguyen TTH, Dudareva N, Pichersky E, Vainstein A (2015). Petunia x hybrida floral scent production is negatively affected by high-temperature growth conditions. Plant Cell Environ.

[30] Ding K, Pei T, Bai Z, Jia Y, Ma P, Liang Z (2017). SmMYB36, a Novel R2R3-MYB Transcription Factor, Enhances Tanshinone Accumulation and Decreases Phenolic Acid Content in Salvia miltiorrhiza Hairy Roots. Sci Rep.

[31] Spitzer-Rimon B, Marhevka E, Barkai O, Marton I, Edelbaum O, Masci T, Prathapani NK, Shklarman E, Ovadis M, Vainstein A. EOBII, a Gene Encoding a Flower-Specific Regulator of Phenylpropanoid Volatiles’ Biosynthesis in Petunia. Plant Cell. 2010;22(6):1961–76. 10.1105/tpc.109.067280 PMC2910970 20543029

[32] Van Moerkercke A, Haring MA, Schuurink RC (2011). The transcription factor EMISSION OF BENZENOIDS II activates the MYB ODORANT1 promoter at a MYB binding site specific for fragrant petunias. Plant J.

[33] Chang S, Puryear J, Cairney J (1993). A simple and efficient method for isolating RNA from pine trees. Plant Mol Biol Report.

[34] Chen SP, Kuo CH, Lu HH, Lo HS, Yeh KW (2016). The Sweet Potato NAC-Domain Transcription Factor IbNAC1 Is Dynamically Coordinated by the Activator IbbHLH3 and the Repressor IbbHLH4 to Reprogram the Defense Mechanism against Wounding. Plos Genetics.

[35] Sherf BA, Navarro SL, Hannah RR, Wood KV (1996). Dual-Luciferase™ Reporter Assay: An Advanced Co-Reporter Technology Integrating Firefly and Renilla Luciferase Assays. Promega Notes Magazine.

