# Supplementary material for: The diurnal emission of floral scent in Oncidium hybrid orchid is controlled by CIRCADIAN CLOCK ASSOCIATED 1 (CCA1) through the direct regulation on terpene synthase
Source: BMC Plant Biol. 2022 Oct 4;22:472. doi: 10.1186/s12870-022-03850-z (PMC9531428; doi:10.1186/s12870-022-03850-z)
Supplement: Supplementary file 1 — Additional file 1: Supplemental Fig. 1. The GC-MS spectrum of floral scent from Oncidium SB flowers in different times. The volatile terpenes, ocimene and linalool display the peaks at RT=11.31 and 12.91 respectively. The profiles decipher floral scents are diurnally emitted highly at 10:00 o’clock and lower at 18:00 o’clock. The GC spectrum showed that there is a circadian rhythm of floral scent emissions. Supplemental Fig. 2. The enzymatic activities assay of TPS. TPS recombinant protein was purified, then fed with the precursor GDP, and reacted at 37℃ for 4hrs. The GC-MS data suggested that the final product was linalool and ocimene. The SPME and GC-MS analysis conditions was described in text. Supplemental Fig. 3. The uncropped image of Fig. 6 was attached. In order to ensure the compliance with the digital image and integrity policies, the uncropped image of Fig. 6 was attached. At two lanes on the right, we increased the concentration of CCA1 when the competitor was added. As the result showed, the fluorescence intensities of free probe decreased. To focus on the interaction between CCA1 and CBS, Figure 6 didn’t show these two lanes. [file 12870_2022_3850_MOESM1_ESM.pdf]

Sample: *Oncidium* Sharry Baby

Sampling Time 10:00

Sampling Time 14:00

Sampling Time 18:00

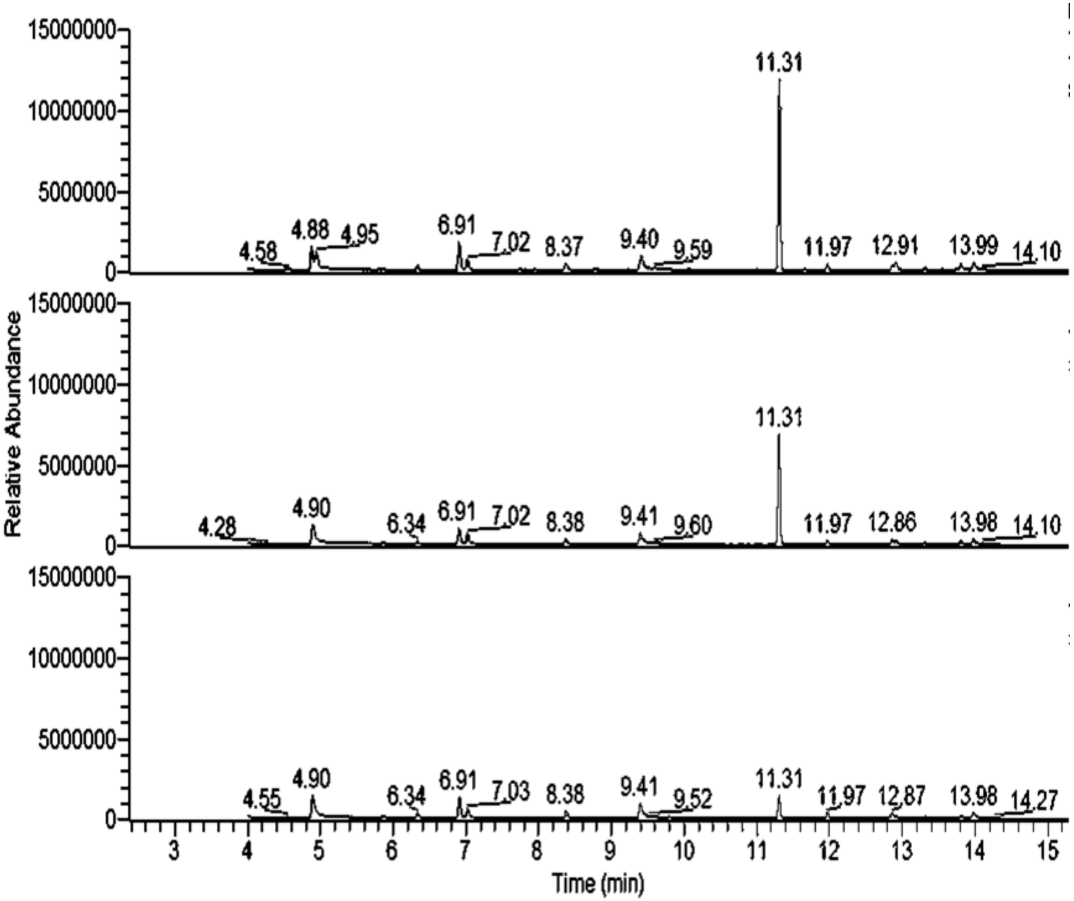


1: 4.88, cyclotrisiloxane
2: 6.91, methoxy-phenyl-oxime
3: 7.02, nonane (internal Standard)
4: 9.40, cyclotetrasiloxane
5: 11.31, β-trans-Ocimene
6: 12.91, linalool
7: 13.99, cyclopentasiloxane

Supplemental fig. 1. the GC-MS spectrum of floral scent from *Oncidium* Sharry Baby flowers in different times.

The volatile terpenes, ocimene and linalool display the peaks at RT=11.31 and 12.91 respectively. The profiles decipher floral scents are diurnally emitted highly at 10:00 o’clock and lower at 18:00 o’clock. The GC spectrum showed that there is a circadian rhythm of floral scent emissions.

Buffer: pH 7.0 phosphate buffer, 1mM $MnCl_2$, 100mM $MgCl_2$
Incubation situation: 37℃, 4hrs

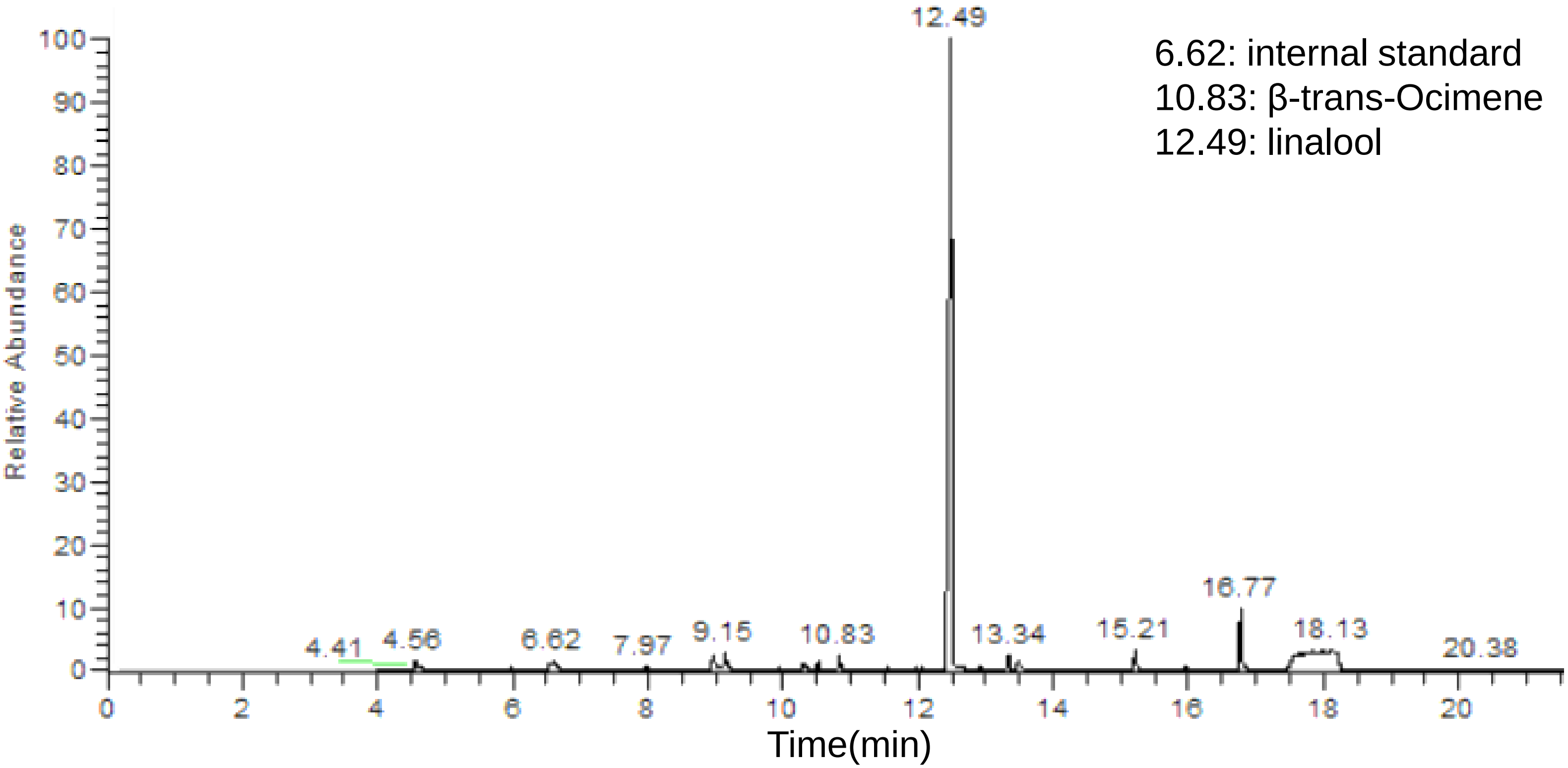


Supplemental fig. 2. the enzymatic activities assay of TPS.
TPS recombinant protein was purified, then fed with the precursor GDP, and reacted at 37℃ for 4hrs. The GC-MS data suggested that the final product was linalool and ocimene. The SPME and GC-MS analysis conditions was described in text.

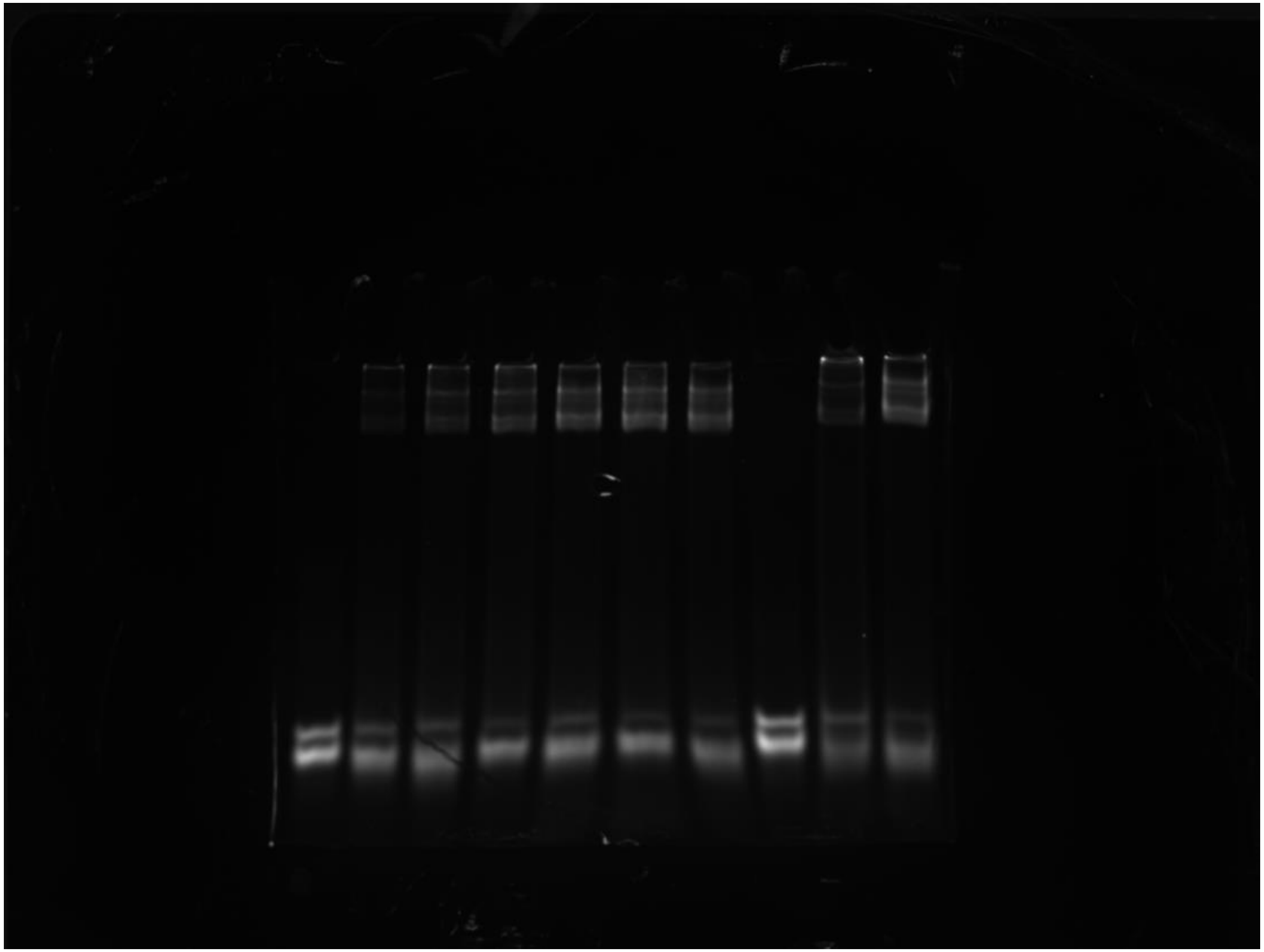

Supplemental fig. 3. The uncropped image of figure 6.
In order to ensure the compliance with the digital image and integrity policies, the uncropped image of figure 6 was attached. At two lanes on the right, we increased the concentration of CCA1 when the competitor was added. As the result showed, the fluorescence intensities of free probe decreased. To focus on the interaction between CCA1 and CBS, fig. 6 didn’t show these two lanes.
